# Reply: Hookah, opium, and tobacco smoking in relation to ESCC

**DOI:** 10.1038/sj.bjc.6604959

**Published:** 2009-03-03

**Authors:** R Malekzadeh, D Nasrollahzadeh, F Kamangar

**Affiliations:** 1 Digestive Diseases Research Center, Tehran University of Medical Sciences, Shariati Hospital, Kargar Street, Tehran 14117, Iran; 2 Medical Epidemiology and Biostatistics, Karolinska Institute, Stockholm, Sweden; 3 Division of Cancer Epidemiology and Genetics, National Cancer Institute, Bethesda, MD, USA

**Sir,**

We thank Dr Chaouachi for his interest in our study. Dr Chaouachi suggests that the association of hookah and oesophageal cancer observed in our study (Nasrollahzadeh *et al*, 2008) may be due to smoking opium, rather than tobacco, by a hookah. Whereas this is a very interesting hypothesis, it is unlikely to explain the results associated with hookah use. The prevalence of hookah use in the study area is low, and only 5% of the study population have used hookah ever. Smoking opium by hookah is not the custom of the local people in North East Iran. In addition, the high sensitivity (93%) of the study questionnaire for detecting opium use significantly decreases any misclassification of exposure to opium (Abnet *et al*, 2004). We think that the conclusion of our study, that every type of tobacco smoking with or without using opium is not a major contributor to oesophageal cancer risk in the study area of Golestan, remains valid.
